# Reliability of Negative Prostate MRI for Biopsy Decision‐Making in the Male Han Chinese Population

**DOI:** 10.1155/proc/1399482

**Published:** 2026-01-13

**Authors:** Fangming Wang, Yan Zhang, Meng Fu, Yuzhe Tang, Haifeng Song, Gang Zhang, Boxing Su, Jianxing Li

**Affiliations:** ^1^ Department of Urology, Tsinghua University Affiliated Beijing Tsinghua Changgung Hospital, Tsinghua University Clinical Institute, Beijing, 102218, China; ^2^ Cardiometabolic Center, State Key Laboratory of Cardiovascular Disease, Fuwai Hospital, National Center for Cardiovascular Diseases, Chinese Academy of Medical Sciences, Peking Union Medical College, Beilishi Road 167, Beijing, 100037, China, pumc.edu.cn

**Keywords:** Chinese, magnetic resonance imaging, prostate cancer

## Abstract

**Background:**

Multiparametric magnetic resonance imaging (mpMRI) has been widely utilized in clinical practice for identifying clinically significant prostate cancer (csPCa). Although mpMRI demonstrates a pooled negative predictive value (NPV) of 90%, additional clinical parameters require evaluation to enhance this metric specifically for the Chinese population—given the rising incidence of PCa in China, as well as ethnic differences in average prostate volume (PV) and chronic prostatitis prevalence that may impact mpMRI’s diagnostic performance.

**Methods:**

A retrospective analysis was conducted on 543 patients who underwent transrectal ultrasound‐guided prostate biopsy at Beijing Tsinghua Changgung Hospital between November 2014 and March 2025. After applying exclusion criteria, 412 patients were enrolled, all of whom had completed prebiopsy mpMRI within 1 month prior to biopsy. Patients were stratified into four groups based on the results of MRI examination and the pathological outcomes of biopsy: MRI (−) PCa (−), MRI (+) PCa (−), MRI (−) PCa (+), and MRI (+) PCa (+) groups. Multivariate logistic regression analyses were used to assess the odd ratios (ORs) of potential predictors for csPCa, comparing the MRI (−) PCa (+) and MRI (−) PCa (−) groups. Receiver operating characteristic curves were generated to analyze the predictive values of total PSA (tPSA), free PSA (fPSA), free‐to‐total (f/t) PSA, PV, PSA density (PSAD), and adjusted PSAD (PSAD^adj^, defined as PSAD × weight) for csPCa in patients with negative MRI.

**Results:**

The patient distribution was as follows: MRI (−) PCa (−) group: 27.9% (115/412), MRI (+) PCa (−) group: 36.9% (152/412), MRI (−) PCa (+) group: 2.4% (10/412), and MRI (+) PCa (+) group: 32.8% (135/412). The NPV of MRI for csPCa was 92%. Multivariate analyses indicated that PV was negatively associated with the presence of csPCa (OR = 0.940, 95% CI: 0.896–0.986, *p* = 0.012), while PSAD and PSAD^adj^ were positively associated with csPCa occurrence (OR = 10.288, 95% CI: 1.569–67.46, *p* = 0.015; OR = 1.027, 95% CI: 1.001–1.053, *p* = 0.043, respectively). For MRI‐negative patients, PV > 55.25 mL (sensitivity = 100%, specificity = 63.2%), PSAD < 0.100 ng/mL^2^ (sensitivity = 100%, specificity = 25.4%), or PSAD^adj^ < 7.24 ng/mL (sensitivity = 100%, specificity = 28.1%) enhanced MRI’s NPV to 100%, while PSAD < 0.205 ng/mL^2^ (sensitivity = 77.8%, specificity = 71.9%) and PSAD^adj^ < 24.97 ng/mL (sensitivity = 55.6%, specificity = 90.4%) improved NPV to 97.6% and 92.6%, respectively.

**Conclusion:**

In Chinese men with negative prostate MRIs, PV > 55.25 mL, PSAD < 0.100 ng/mL^2^, or PSAD^adj^ < 7.24 ng/mL may elevate mpMRI’s NPV from 92% to 100%, enabling safe avoidance of unnecessary biopsies. Prospective multicenter validation is required to confirm these findings.

## 1. Introduction

Prostate cancer (PCa) is the second‐most frequent cancer and the fifth leading cause of cancer death among men worldwide [1]. Although prostate‐specific antigen (PSA) screening is commonly employed for the detection of PCa, its low specificity frequently leads to unnecessary prostate biopsies and the overdiagnosis of nonclinically significant PCa (non‐csPCa) [2], creating an urgent need for more precise secondary screening tools.

Multiparametric magnetic resonance imaging (mpMRI) using the Prostate Imaging Reporting and Data System (PI‐RADS) has become a key tool for PCa detection and risk stratification [3, 4]. A negative MRI (PI‐RADS ≤ 2) indicates a low probability of the presence of csPCa [5]. Despite its ability to improve csPCa detection and reduce overdiagnosis, mpMRI has limitations. It poses a risk of false‐negative results, potentially causing csPCa underdetection. One meta‐analysis demonstrated that the negative predictive value (NPV) of MRI for csPCa was more than 90% across populations although heterogeneity in the NPV estimates in different institutions was observed [6]. Another meta‐analysis reported that the NPV of mpMRI for PCa was 85%–95% [7]. However, most studies reporting MRI’s NPV for csPCa were from Western countries, and there was a lack of studies on Chinese populations [6]. Besides, PCa incidence and mortality have rapidly increased in China [8], and NPV significantly decreased when cancer prevalence increased [7]. This highlights the need to investigate the diagnostic value of negative mpMRI in Chinese men, as institutional‐specific data can inform clinical decision‐making.

Given that approximately 10% of csPCa may be missed if biopsies are omitted in mpMRI‐negative patients [6, 7], some researchers have explored clinical parameters to predict which men are likely to harbor csPCa despite negative MRI results, such as the commonly used PSA density (PSAD), digital rectal examination results [9–11]. Chinese men differ from Western populations in terms of average prostate volume and prostatitis prevalence [12–14]. For instance, a larger average prostate volume lowers the relative burden of csPCa, whereas chronic prostatitis can induce false‐positive MRI signals, thereby interfering with the interpretation of NPV. Further exploration of clinical parameters is therefore necessary to guide biopsy decisions in mpMRI‐negative Chinese patients.

This study analyzed the distribution of mpMRI results and PCa diagnoses in Chinese biopsy patients, evaluating and comparing the efficacy of clinical parameters to enhance mpMRI’s NPV. The goal was to provide evidence for safe biopsy omission in men with elevated PSA but negative mpMRI, reducing unnecessary procedures.

## 2. Patients and Methods

### 2.1. Study Subjects

**Figure 1:**
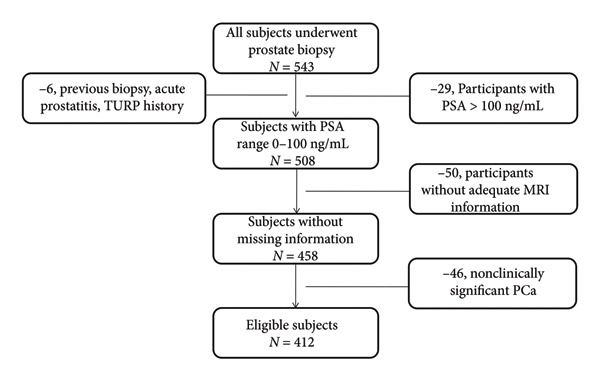
The enrollment process for subjects who underwent prostate biopsy in the study. Initially, a total of 543 subjects received prostate biopsies. First, six patients with a history of previous prostate biopsy or TURP or acute prostatitis and 29 patients with a PSA level > 100 ng/mL were excluded. After this step, 508 subjects with a PSA range of 0–100 ng/mL remained. Next, 50 subjects without adequate MRI information were excluded from these 508 subjects, resulting in 458 subjects with no missing information. Finally, 46 subjects with nonclinically significant PCa were excluded from the 458 subjects. Eventually, 412 eligible subjects were enrolled. TURP: transurethral resection of the prostate; PSA: prostate‐specific antigen; MRI: magnetic resonance imaging; PCa: prostate cancer.

A retrospective review was conducted on 543 patients who underwent transrectal ultrasound‐guided prostate biopsy at Beijing Tsinghua Changgung Hospital between November 2014 and March 2025. The inclusion criteria were as follows: (1) first‐time prostate biopsy; (2) prebiopsy mpMRI completed within 1 month of biopsy (to ensure consistency between imaging and biopsy status); and (3) complete clinicopathological data, including age, body mass index (BMI), serum tPSA and fPSA levels (measured within 2 weeks of biopsy), prostate volume (assessed via mpMRI), and biopsy pathology results. The exclusion criteria were as follows: (1) acute prostatitis, prostatic massage, digital rectal examination, or cystoscopy within 2 weeks prior to PSA testing; (2) tPSA > 100 ng/mL; (3) missing or incomplete mpMRI data; (4) non‐csPCa (International Society of Urological Pathology [ISUP] grade ≤ 1, to focus on clinically relevant disease); and (5) prior surgical treatment for benign prostatic hyperplasia (BPH). After exclusions, 412 patients were eligible for analysis (see Figure 1 for the enrollment flowchart).

### 2.2. Collection of Patients’ Clinicopathological Data

All clinicopathological data were extracted from the hospital information system, including age, BMI, serum tPSA and fPSA levels, prostate volume, PSAD, adjusted PSA density (PSAD^adj^), mpMRI findings, and biopsy results (number of positive cores and ISUP grade). BMI was calculated as the reported weight (in kilograms) divided by the square of height (in meters). Prostate volume was estimated by measuring the prostate dimensions from 3D T2‐weighted MRI images, using the following formula: transverse diameter × anteroposterior diameter × craniocaudal diameter × 0.52. The ratio of free PSA to total PSA was denoted as f/t PSA. PSAD was determined by dividing the serum tPSA value by the prostate volume. PSAD^adj^ was calculated by multiplying PSAD by body weight.

### 2.3. MRI Acquisition and Interpretation

All patients routinely underwent MRI examination with a 3.0 T scanner before biopsy. The MRI inquisition protocol included T1‐weighted imaging (T1WI), T2‐weighted imaging (T2WI), diffusion‐weighted imaging (DWI), and dynamic contrast‐enhanced MR imaging (DCE‐MRI). The apparent diffusion coefficient (ADC) maps were generated from DWI with *b* values of 0, 100, 1000, and 2000 s/mm^2^. mpMRI images were assessed on a PACS workstation using the PI‐RADS version 2.0 (before 2019) and version 2.1 (2019 or later), respectively, and was interpreted by two specialized genitourinary radiologists at our center. Any discrepancies between radiologists were resolved through discussion. According to both versions, MRI was considered negative with a score of ≤ 2, and positive with a score of ≥ 3.

### 2.4. Prostate Biopsy

**Figure 2:**
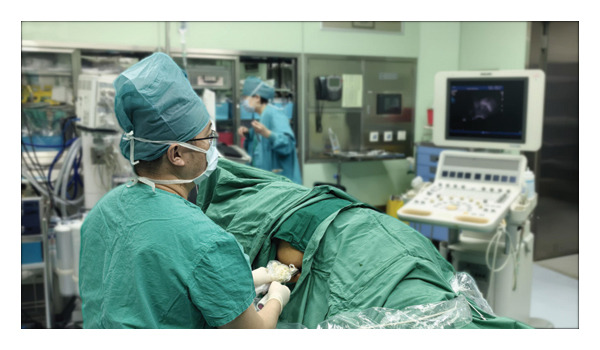
The surgical scene of an ultrasound‐guided transrectal biopsy performed by Dr. Fangming Wang in our hospital. The patient is positioned in the left‐lateral decubitus position to facilitate access to the rectum. After sterilizing the perianal and rectal regions, we administered local anesthesia into the rectal wall to numb the area, reducing the patient’s discomfort. Using a transrectal ultrasound probe, we visualized the prostate gland in real‐time, carefully mapping its contours and identifying any suspicious areas. The biopsy needle is then carefully inserted through the rectal wall under the guidance of the ultrasound. With precise and steady hand movements, we advanced the needle into the targeted regions of the prostate. Small tissue samples are retrieved from multiple sites within the gland, typically from different lobes and zones, to ensure a comprehensive assessment. The patient’s vital signs are also continuously observed by the nursing staff to ensure their stability. Once the required number of tissue samples has been obtained, the needle is carefully withdrawn, and the procedure is concluded. The tissue samples are then promptly sent to the pathology laboratory for microscopic examination to aid in the diagnosis of prostate‐related conditions.

As illustrated in Figure 2, all biopsies were performed under local anesthesia using cognitive fusion technique combining mpMRI findings and transrectal ultrasound guidance (Philips, HD15 ultrasound system). Specifically, in addition to obtaining 12 systematic cores (the inner and outer sides of the left and right prostatic apex, body, and fundus), we targeted 1‐2 cores at lesions through combined of mpMRI‐identified lesions and hypoechoic lesions detected on real‐time ultrasound observations. Subsequently, the collected specimens were labeled, fixed in formalin, and transferred to the pathology department for diagnostic evaluation.

### 2.5. Statistical Analysis

Continuous variables were presented as mean ± standard deviation (SD) for normally distributed data or median (interquartile range) for nonnormally distributed data. Categorical variables were expressed as counts (percentages). Between‐group comparisons of continuous variables were performed using one‐way analysis of variance (ANOVA) or unpaired *t*‐tests (parametric data) and Kruskal–Wallis or Mann–Whitney *U* tests (nonparametric data). Patients were stratified into four groups based on mpMRI results and biopsy pathology: MRI (−) PCa (−), MRI (+) PCa (−), MRI (−) PCa (+), and MRI (+) PCa (+). Clinical variables (age, BMI, prostate volume, PSA‐derived parameters, and number of positive cores) were compared across groups. Multivariate logistic regression analysis was conducted to assess the predictive ability of all PSA‐derived parameters and prostate volume for diagnosing PCa within four distinct models. Model 1 incorporated variables such as age, BMI, tPSA, fPSA, and prostate volume; Model 2 comprised variables such as age, BMI, f/tPSA, and prostate volume; Model 3 included age, BMI, and PSAD as variables; and Model 4 consisted of age and PSAD^adj^ as variables. No additional weighting was applied to parameters in the multivariate logistic regression models. The contribution of each parameter to PCa prediction is reflected by the odds ratio (OR), which quantifies the independent association between the parameter and PCa (adjusted for other variables). ORs were calculated via maximum likelihood estimation. Receiver operating characteristic (ROC) curves were plotted, and the area under the ROC curve (AUC) was calculated to evaluate and compare the predictive values of prostate volume and PSA‐derived parameters in detecting PCa among patients with negative MRI findings. The optimal cutoff value for each parameter was determined from the corresponding ROC curves, and both the sensitivity and specificity for predicting PCa were calculated. The Youden index, defined as sensitivity + specificity − 1, was employed to identify the most appropriate cutoff point. Pairwise AUC differences were tested for significance using DeLong’s test. All statistical tests were two‐sided, with *p* < 0.05 considered statistically significant. Analyses were conducted using SPSS 22.0 (SPSS Inc., Chicago, IL, USA), GraphPad Prism 8, and R 4.2.2 (*R* Foundation for Statistical Computing, Vienna, Austria).

## 3. Ethics Statement

This study was approved by the Ethical Committee of Beijing Tsinghua Changgung Hospital (Approval No. 2024‐012‐01) and conducted in accordance with local legislation and institutional guidelines. Informed consent was waived due to the retrospective nature of the study, with data extracted from the hospital’s electronic medical record system.

## 4. Results

### 4.1. Comparison of Clinicopathological Parameters Among Groups Stratified by MRI and Biopsy Pathological Results in the Whole Cohort

**Figure 3:**
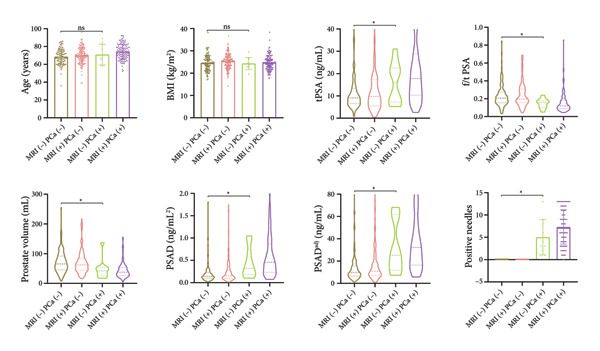
Comparison of age, BMI, PSA‐derived parameters, prostate volume, and positive needles among the MRI (−) PCa (−) (*n* = 115), MRI (+) PCa (−) (*n* = 152), MRI (−) PCa (+) (*n* = 10), and MRI (+) PCa (+) (*n* = 135) groups. Data are expressed as mean ± SD, or median (interquartile range). The median with interquartile range is shown in a violin plot (the dotted line). Statistical significance was determined using an unpaired *t*‐test or the Mann–Whitney *U* test. The figures were drawn using GraphPad Prism 8 software (GraphPad Software Inc., La Jolla, CA, USA). ns, not significant; ^∗^ *p* < 0.05. MRI: magnetic resonance imaging; PCa: prostate cancer; BMI: body mass index; tPSA: total prostate‐specific antigen; f/t PSA: free‐to‐total PSA ratio; PSAD: prostate‐specific antigen density; PSAD^adj^: adjusted PSAD.

The clinicopathological characteristics, including age, BMI, prostate volume, PSA derivative parameters, and positive needle findings, of the MRI (−) PCa (−), MRI (+) PCa (−), MRI (−) PCa (+), and MRI (+) PCa (+) groups are exhibited in Table 1. Among the 412 eligible men who underwent biopsy, 145 were diagnosed with csPCa, resulting in a positive biopsy rate of 35.2%. The distribution of the subjects was as follows: MRI (−) PCa (−) (27.9%, 115/412), MRI (+) PCa (−) (36.9%, 152/412), MRI (−) PCa (+) (2.4%, 10/412), and MRI (+) PCa (+) (32.8%, 135/412). The percentage of negative MRI was 30.3% (115 + 10/412), and the overall diagnostic performance of MRI for csPCa was sensitivity = 93.1% (135/135 + 10), specificity = 43.8% (115/115 + 152), NPV = 92.0% (115/115 + 10), and positive predictive value (PPV) = 47.1% (135/287). There was significant difference among the four groups in all the listed clinicopathological parameters except BMI. Focused on those patients with negative MRI including MRI (−) PCa (+) and MRI (−) PCa (−) subgroups, no significant differences were found in age and BMI between the two subgroups. To identify predictors of csPCa in MRI‐negative patients, we compared clinicopathological parameters between MRI (−) PCa (−) and MRI (−) PCa (+) groups. The tPSA, PSAD, PSAD^adj^, and positive needles were significantly higher in the MRI (−) PCa (+) group than in the MRI (−) PCa (−) group (*p* < 0.05), whereas f/t PSA and prostate volume were significantly lower in the MRI (−) PCa (+) group than in the MRI (−) PCa (−) group (*p* < 0.05) (Table 1 and Figure 3). Collectively, levels of PSA‐derived parameters, prostate volume, and number of positive needles were significantly different between the MRI (−) PCa (+) and MRI (−) PCa (−) groups.

**Table 1:** Demographic and clinical characteristics of subjects who underwent prostate biopsy based on MRI and biopsy pathological results and comparison of clinicopathological characteristics between MRI (−) PCa (−) and MRI (−) PCa (+) subgroups.

	MRI (−) PCa (−) *n* = 115	MRI (+) PCa (−) *n* = 152	MRI (−) PCa (+) *n* = 10	MRI (+) PCa (+) *n* = 135	*p* value
Age (years)	68.6 ± 8.4	69.9 ± 8.8	70.9 ± 11.7	74.07 ± 8.4	0.432
BMI (kg/m^2^)	24.5 ± 3.5	25.3 ± 3.4	24.3 ± 2.9	24.7 ± 3.4	0.865
tPSA (ng/mL)	9.1 (6.6–13.4)	9.8 (5.4–17.2)	16.1 (7.3–22.5)	17.8 (10.4–28.9)	**0.003**
f/t PSA ratio	0.21 (0.15–0.28)	0.20 (0.16–0.31)	0.16 (0.10–0.18)	0.12 (0.09–0.18)	**0.008**
Prostate volume (mL)	65.7 (45.5–92.0)	62.4 (44.5–83.4)	42.0 (25.2–53.0)	37.7 (26.6–53.8)	**< 0.001**
PSAD (ng/mL^2^)	0.14 (0.10–0.22)	0.15 (0.09–0.28)	0.32 (0.18–0.75)	0.46 (0.23–0.87)	**< 0.001**
PSAD^adj^ (ng/mL)	9.72 (6.54–15.76)	10.96 (7.18–18.88)	25.18 (10.63–61.73)	32.26 (16.40–62.83)	**< 0.001**
Positive needles (*n*)	0	0	5.0 ± 3.9	7.3 ± 3.8	**< 0.001**

*Note:* Normally distributed continuous variables are presented as mean ± SD (analyzed by independent samples *t*‐test); nonnormally distributed continuous variables are presented as median (IQR) (analyzed by Mann–Whitney *U* test). The bold value indicated statistical significance. *p* values represent the comparison result of MRI (−) PCa (−) and MRI (−) PCa (+) subgroups, and *p* values < 0.05 indicate statistically significant differences. MRI (−): negative multiparametric magnetic resonance imaging; PCa (−)/PCa (+): negative/positive prostate cancer; f/t PSA ratio: free/total prostate‐specific antigen ratio; PSAD^adj^: adjusted prostate‐specific antigen density.

Abbreviations: IQR, interquartile range; PSAD, prostate‐specific antigen density; SD, standard deviation, tPSA, total prostate‐specific antigen.

### 4.2. Retrospective Re‐evaluation of MRI Images in MRI (−) PCa (+) Cases

We retrospectively re‐evaluated the mpMRI images of the 10 MRI (−) PCa (+) cases with two senior genitourinary radiologists (blinded to pathology results). The re‐evaluation focused on T2WI hypointensity, DWI restriction, and DCE enhancement (key PI‐RADS 2.1 criteria). Results showed that eight cases had subtle, focal hypointensities on T2WI (located in the peripheral zone) but no corresponding DWI restriction or DCE enhancement (thus not meeting PI‐RADS ≥ 3) and two cases had no obvious abnormal signals on all sequences. No missed findings that would change the original PI‐RADS ≤ 2 classification were identified, indicating that the false‐negative results were due to the inherent limitations of mpMRI rather than interpretive errors.

### 4.3. Correlations of PSA Derivative Parameters With the Presence of csPCa in MRI‐Negative Cohort

We performed multivariate logistic regression analysis to evaluate the correlations between PSA‐related parameters and other clinical variables with PCa occurrence in those biopsy patients with negative MRI findings. As shown in Table 2, in Model 1, we revealed that prostate volume was significantly and negatively correlated with the presence of PCa (OR = 0.940, 95% CI: 0.896–0.986, *p* = 0.012), whereas other parameters including age, BMI, tPSA, and fPSA were not independently correlated with the presence of PCa. In Model 2, we changed PSA‐related parameters to f/t PSA and found that prostate volume remained significantly correlated with the PCa presence after adjusting for the mentioned confounding factors as in Model 1 (OR = 0.942, 95% CI: 0.898–0.989, *p* = 0.016). In Models 3 and 4, we demonstrated that PSAD and PSAD^adj^ were significantly and positively correlated with PCa occurrence (OR = 10.288, 95% CI: 1.569–67.46, *p* = 0.015; OR = 1.027, 95% CI: 1.001–1.053,*p* = 0.043, respectively). Next, we explored the predictive value of the identified parameters including prostate volume, PSAD, and PSAD^adj^ in MRI‐negative patients by plotting ROC curves.

**Table 2:** Multivariate analysis to compare the independent correlations between prostate volume or different PSA‐derived parameters and the presence of csPCa in prostate biopsied patients with negative MRI.

Models	Variables	Multivariate mode		
		OR	95% CI	*p* value
1	Age	1.063	0.978–1.155	0.149
	BMI	1.056	0.853–1.307	0.616
	tPSA	1.085	0.980–1.201	0.118
	fPSA	0.633	0.294–1.365	0.244
	Prostate volume	0.940	0.896–0.986	**0.012**

2	Age	1.060	0.977–1.149	0.161
	BMI	1.039	0.845–1.278	0.717
	f/tPSA	0.000	0.000–9.563	0.129
	Prostate volume	0.942	0.898–0.989	**0.016**

3	Age	1.020	0.942–1.104	0.626
	BMI	0.964	0.781–1.190	0.734
	PSAD	10.288	1.569–67.46	**0.015**

4	Age	1.022	0.943–1.108	0.596
	PSAD^adj^	1.027	1.001–1.053	**0.043**

*Note:* Multivariate regression models are shown. The bold value indicated statistical significance. The dependent variable was the presence of PCa. tPSA, total PSA; fPSA, free PSA; PSAD^adj^, adjusted PSAD; 95% CI: 95% confidence interval; csPCa, clinically significant prostate cancer; f/t PSA, free/total prostate‐specific antigen ratio.

Abbreviations: BMI, body mass index; MRI, magnetic resonance imaging; OR, odds ratio; PSA, prostate‐specific antigen; PSAD, prostate‐specific antigen density.

### 4.4. The Diagnostic Performance of Prostate Volume, PSAD, and PSAD^adj^ in MRI‐Negative Patients

**Figure 4:**
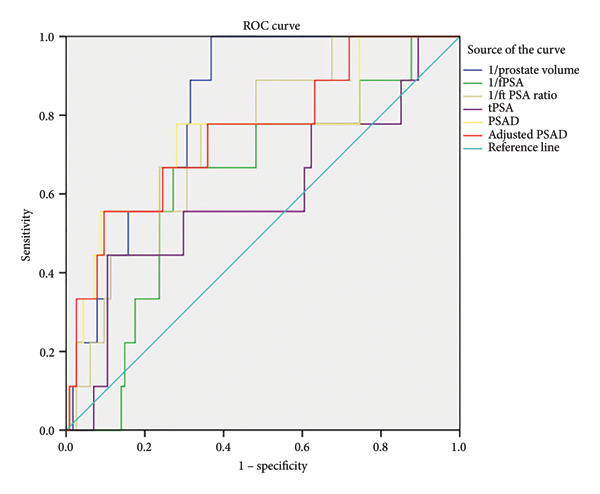
Comparison of the diagnostic efficacy of prostate volume, fPSA, f/t PSA, tPSA, PSAD, and adjusted PSAD for PCa in patients with negative MRI results. ROC: receiver operating characteristic; 1/prostate volume: the reciprocal of prostate volume; 1/fPSA: the reciprocal of free prostate‐specific antigen; 1/ft PSA: the reciprocal of free‐to‐total PSA ratio; tPSA, total PSA; PSAD, prostate‐specific antigen density; PCa: prostate cancer; MRI: magnetic resonance. imaging.

The predictive values of tPSA, fPSA, f/tPSA, prostate volume, PSAD, and PSAD^adj^ for PCa were analyzed by determining the AUC for each of the tests in patients with negative MRI (Figure 4). The AUC values for tPSA, fPSA, f/tPSA, prostate volume, PSAD, and PSAD^adj^ were 0.594, 0.632, 0.740, 0.820, 0.750, and 0.756, respectively. Based on ROC analysis, for prostate volume, a threshold of 55.25 mL yielded a Youden’s index maximum of 0.632, which was higher than those Youden’s index values of PSAD, PSAD^adj^, and other parameters. Besides, the sensitivity and specificity of prostate volume was 100% and 63.2%, respectively (Table 3). Youden’s index of PSAD, a parameter routinely used for PCa diagnosis, was 0.497. The corresponding cutoff value was 0.205, with a sensitivity of 77.8% and a specificity of 71.9%. Moreover, when the cutoff value of PSAD was decreased to 0.15, the sensitivity remained at 77.8%, while the specificity dropped to 52.6%. When further decreased to 0.10, the sensitivity of PSAD reached 100%, but the specificity decreased to 25.4%. Similar to the situation of PSAD, Youden’s index of PSAD^adj^ was 0.460. The corresponding cutoff value was 24.97, yielding a sensitivity of 55.6% and a specificity of 90.4%. Furthermore, upon reducing the cutoff value of PSAD^adj^ to 7.242, the sensitivity ascended to 100%, yet the specificity declined to 28.1%. It was noteworthy that the AUC of PSAD^adj^ was the largest among all PSA‐related parameters. More importantly, prostate volume > 55.25 mL, PSAD < 0.205 ng/mL^2^, PSAD < 0.150 ng/mL^2^, PSAD < 0.100 ng/mL^2^, PSAD^adj^ < 24.97 ng/mL, and PSAD^adj^ < 7.24 ng/mL have been shown to enhance MRI’ NPV to 100%, 97.6%, 96.8%, 100%, 92.6%, and 100%, respectively. For MRI‐negative patients (*n* = 125): (1) PV > 55.25 mL (*n* = 42) avoided 42 biopsies (33.6% of MRI‐negative patients) with 0 missed csPCa (100% NPV); (2) PSAD < 0.100 ng/mL^2^ (*n* = 31) avoided 31 biopsies (24.8%) with 0 missed csPCa; (3) PSAD^adj^ < 7.24 ng/mL (*n* = 35) avoided 35 biopsies (28.0%) with 0 missed csPCa; and (4) PSAD < 0.205 ng/mL^2^ (*n* = 58) avoided 58 biopsies (46.4%) but missed 2 csPCa (8.0% missing rate, NPV = 97.6%). Table 4 compares the AUCs of clinical parameters for predicting csPCa in MRI‐negative patients. Prostate volume had the highest AUC, significantly higher than tPSA (*p* = 0.008) and fPSA (*p* = 0.021), but not different from f/tPSA (*p* = 0.152), PSAD (*p* = 0.214), or PSAD^adj^ (*p* = 0.256). tPSA (lowest AUC) and fPSA had significantly lower AUCs than f/tPSA, PSAD, and PSAD^adj^ (all *p* < 0.05), while f/tPSA, PSAD, and PSAD^adj^ showed no AUC differences (all *p* > 0.05). In summary, the specificity and NPV of prostate volume was superior to PSA‐related parameters despite its slightly poor sensitivity for those patients with negative MRI, indicating that prostate volume has the highest clinical value to avoid unnecessary prostate biopsy.

**Table 3:** Diagnostic efficacy of PSA‐derived parameters and prostate volume for csPCa detection in men with negative MRI.

Predictors	AUC	95% CI	*p* value	Cutoff	Sensitivity (%)	Specificity (%)	Youden’s index	NPV (%)
tPSA	0.594	0.379–0.808	0.351	20.48	44.4	89.5	0.339	94.4

fPSA	0.632	0.453–0.810	0.190	1.34	66.7	72.8	0.395	95.5

f/tPSA	0.740	0.596–0.884	**0.017**	0.175	77.8	65.8	0.436	97.4

Prostate volume	0.820	0.723–0.917	**0.001**	55.25	100	63.2	0.632	100

PSAD	0.750	0.563–0.938	**0.013**	0.205	77.8	71.9	0.497	97.6
				0.150	77.8	52.6	0.304	96.8
				0.100	100	25.4	0.254	100

PSAD^adj^	0.756	0.584–0.929	**0.011**	24.97	55.6	90.4	0.460	92.6
				7.24	100	28.1	0.281	100

*Note:* The bold value indicated statistical significance. f/tPSA: free/total PSA; PSAD: free prostate‐specific antigen density; csPCa, clinically significant prostate cancer; PSAD^adj^: adjusted PSAD.

Abbreviations: AUC, area under curve; fPSA, free prostate‐specific antigen; MRI, magnetic resonance imaging; NPV, negative predictive value; PSA, prostate‐specific antigen; tPSA, total prostate‐specific antigen.

**Table 4:** Comparison of AUC values of different clinical parameters for predicting csPCa in MRI‐negative patients.

Parameter	AUC (95% CI)	Comparison with other parameters	*p* value
Prostate volume	0.820 (0.723–0.917)	vs. tPSA	**0.008**
		vs. fPSA	**0.021**
		vs. f/tPSA	0.152
		vs. PSAD	0.214
		vs. PSAD^adj^	0.256

tPSA	0.594 (0.379–0.808)	vs. fPSA	0.483
		vs. f/tPSA	**0.012**
		vs. PSAD	**0.009**
		vs. PSAD^adj^	**0.007**

fPSA	0.632 (0.453–0.810)	vs. f/tPSA	**0.043**
		vs. PSAD	**0.038**
		vs. PSAD^adj^	**0.031**

f/tPSA	0.740 (0.596–0.884)	vs. PSAD	0.785
		vs. PSAD^adj^	0.712

PSAD	0.750 (0.563–0.938)	vs. PSAD^adj^	0.896

PSAD^adj^	0.756 (0.584–0.929)	—	—

*Note:* Statistical comparisons of AUC values were performed using DeLong’s test. The bold value indicated statistical significance. *p* values < 0.05 indicate statistically significant differences between parameters. AUC: area under the receiver operating characteristic curve; f/tPSA: free/total prostate‐specific antigen ratio; PSAD^adj^: adjusted PSAD.

Abbreviations: CI, confidence interval; fPSA, free prostate‐specific antigen; PSAD, prostate‐specific antigen density; tPSA, total prostate‐specific antigen.

## 5. Discussion

Our study reported that, among 412 patients who had a prebiopsy MRI, a negative MRI was found in 125 patients (30.3%), among whom 8% had csPCa on biopsy. Thus, MRI had an overall NPV of 92% for csPCa. Prostate volume > 55.25 mL, PSAD < 0.100 ng/mL^2^, or PSAD^adj^ < 7.24 ng/mL could skip biopsy in men who had a negative MRI. While larger cohorts for MRI‐based PCa diagnosis exist in non‐Chinese populations (e.g., [3, 15]), data from Chinese populations remain limited, especially regarding population‐specific parameters (e.g., PV, PSAD) that may modify MRI’s NPV. Our single‐center cohort of 412 patients addresses this gap by providing Chinese‐specific thresholds for biopsy decision‐making, which cannot be directly extrapolated from Western studies due to differences in average PV and chronic prostatitis prevalence. To our knowledge, this is the first study to systematically combine prostate volume and PSA‐related parameters to optimize biopsy decisions in mpMRI‐negative Chinese patients, offering a practical tool to reduce unnecessary procedures and associated patient discomfort and healthcare costs.

The use of mpMRI has become an important modality in the evaluation of PCa, helping to detect csPCa and guide subsequent biopsy procedures. The incidence rate of negative MRI for PCa shows substantial variation. Ahmed and his colleagues reported that 27% of cases had negative MRI results when the mean PSA level was 7.1 ng/m^2^ [3], and Leest and associates indicated that 49% of patients had negative MRI findings with median PSA level of 6.4 ng/mL [15]. Arulraj et al. reported that the incidence of negative MRI was only 9% with the median PSA level of 11.6 ng/mL in the Indian population [9]. Our study reported that negative MRIs was found in 125 patients among 412 patients (30.3%), with the median PSA of 11.15 ng/mL. This value of 30.3 fell within the range of previously reported data (27%, 49%) [15] and was lower than the average incidence rate of 33% documented in the existing literature on PCa diagnosis [16, 17]. Two potential factors may account for the variability in the incidence of negative MRI for PCa. First, chronic prostatitis can present radiologically similar to malignancy on MRI, often leading to elevated PI‐RADS scores. Consequently, patients with prostatitis may receive positive MRI results despite the absence of cancer. The incidence of chronic prostatitis in Chinese men undergoing biopsies may have a higher incidence of chronic prostatitis than Western counterparts but lower incidence than Indian counterparts. Second, significant heterogeneity exists in MRI reporting practices. MRI scans in our hospital were consistently evaluated by two experienced radiologists. However, defensive reporting bias may be present among radiologists. To minimize the risk of overlooking PCa in equivocal cases, radiologists may be inclined to report a higher proportion of MRIs as positive.

In our study, the incidence of csPCa among men with negative MRI was 8%, which was comparable to previously reported in the literature [3, 7, 18]. However, some research has also identified higher incidences of csPCa (more than 30%) in MRI‐negative patients [19, 20]. Furthermore, research comparing MRI with histopathological examination of postradical prostatectomy (RP) specimens has indicated that MRI may fail to detect csPCa. Branger and colleagues investigated RP specimens from 101 men with negative MRI findings and found that 61.3% of them had adverse pathological outcomes [21]. Similarly, Kim et al. reported 117 (59.7%) with GS 3 + 4 and 44 (22.4%) with GS ≥ 4 + 3, while the rates of favorable PCa and insignificant cancer were only 14.3% and 10.2%, respectively [22]. These studies show that a significant proportion of men with negative MRI harbored csPCa, which might be attributed to the interobserver variability.

Our study showed that MRI had a 92% NPV in Chinese men, meaning that 8% of negative MRI results were inaccurate. Therefore, negative MRIs should not be used alone to skip biopsies in Chinese men, indicating a need for additional screening tools. Although the EAU guidelines recommend a 90% NPV threshold for biopsy omission [23], we identified population‐specific thresholds that enhance MRI’s NPV to 100% in Chinese men; these thresholds address unmet needs not covered by Western guidelines. We explored the ORs of prostate volume and PSA‐related parameters for the prediction of PCa presence in MRI‐negative patients using multivariate logistic analysis. From the four multivariate predicting models we built, we proved that prostate volume was a negative factor that was independently correlated with PCa occurrence, whereas PSAD and PSAD^adj^ were negative factors that were independently correlated with the PCa presence. The following reasons can account for our finding that prostate volume was inversely related to the PCa detection rate in MRI‐negative patients: (1) As the prostate volume grows, the relative amount of PCa tissue diminishes, leading to a reduction in the PCa detection rate and (2) like the “big bang theory” in physics, as the prostate gradually grows and expands from middle to old age, the potentially lesion zone could grow larger simultaneously. The large volume has an amplification effect on the potential lesion zone. Therefore, in larger prostates with no visible MRI lesions, the probability of PCa is much lower.

Notably, the csPCa missed in our MRI‐negative cohort was predominantly small‐volume ISUP Grade 2 lesions, which typically exhibit indolent biological behavior. Branger et al. [21] analyzed RP specimens from MRI‐negative patients and found that 73% (27/37) of missed csPCa were small GG2 lesions, with no evidence of extracapsular extension or lymph node involvement, suggesting limited clinical impact and supporting the safety of biopsy omission in patients meeting our established thresholds.

We further explored the sensitivity, specificity, and NPV of prostate volume and various PSAD and PSAD^adj^ thresholds for PCa detection in Chinese men with negative MRI. In the present study, the AUC value of 0.820 for prostate volume was the highest among all parameters, and a cutoff of 55.25 for prostate volume will produce 100% sensitivity, 63.2% specificity, and 100% NPV, which has great translational value and needs further external validation. PSAD is a commonly used parameter to enhance MRI’s NPV in the literature [9, 10, 24]. Falagario et al. found that using PI‐RADS < 3 and PSAD < 0.15 sequentially gave a 95% NPV [24]. Sandlow S et al. found that using PSAD < 0.09 for PI‐RADS 1‐2 has an NPV and sensitivity > 90% to detect csPCa in Black men [10]. Arulraj et al. demonstrated that the optimal threshold of PSAD for the diagnosis of csPCa was 0.25 ng/mL/cc (Youden’s index). The sensitivity, specificity, and NPV for a PSAD of 0.25 ng/mL/cc in predicting csPCa were 76%, 82%, and 87%, respectively [9]. In our data, the optimal threshold of PSAD for the diagnosis of csPCa was 0.205 ng/mL^2^ (Youden’s index) and produced 77.8% sensitivity, 71.9% specificity, and 97.6% NPV. The commonly used PSAD < 0.15 threshold had 77.8% sensitivity, 52.6% specificity, and 96.8% NPV. PSAD < 0.10 improves sensitivity and NPV for detecting csPCa in Chinese men with negative lesions to 100%, despite reduced specificity (71.9%–52.6% to 25.4%). Our proposed strategy involves biopsying all MRI‐positive lesions, deferring biopsy in MRI‐negative patients if PSAD < 0.10. Comparatively, using PSAD < 0.15 or 0.205 in our proposed strategy had lower NPV, at 96.8% and 97.6%, respectively, suggesting that Chinese men with negative MRIs may need another biomarker to safely defer biopsy. The predictive value of PSAD combined with PI‐RADS for PCa has been reported in Western populations [10, 24]. Our study extends these findings by validating this combination in Chinese men and identifying a lower PSAD threshold that achieves 100% NPV. PSAD^adj^ was a concept of adjusted PSAD (calculated by PSAD × weight), which was proposed by us last year to solve the problem of blood volume variations when the PSAD value was used for the PCa diagnosis [25]. The PSAD^adj^ threshold of 24.97 had 55.6% sensitivity, 90.4% specificity, and 92.6% NPV, while PSAD^adj^ < 7.24 improves sensitivity and NPV to 100%, despite reduced specificity (90.4%–28.1%). We acknowledge that 100% NPV is not a universal target for PCa diagnostics, as it may require accepting lower specificity (e.g., 25.4% for PSAD < 0.100 ng/mL^2^) and fewer avoided biopsies. However, our thresholds (PV > 55.25 mL, PSAD < 0.100 ng/mL^2^, PSAD^adj^ < 7.24 ng/mL) achieved a favorable balance: They reduced unnecessary biopsies by 24.8%–33.6% in MRI‐negative patients while maintaining 100% NPV (no csPCa missed). For scenarios prioritizing higher biopsy avoidance, PSAD < 0.205 ng/mL^2^ (46.4% reduction) may be acceptable, although it carries an 8.0% missing rate—with missed lesions being ISUP Grade 2 tumors of low clinical significance. This balance allows clinicians to tailor thresholds to patient preferences (e.g., biopsy avoidance vs. fear of missed cancer).

This study has several limitations. First, the retrospective design may introduce selection bias. To minimize selection bias, we applied strict inclusion/exclusion criteria and standardized data extraction from the hospital’s electronic medical record system. Additionally, MRI interpretations were conducted by two specialized genitourinary radiologists, with discrepancies resolved via consensus, reducing interpretive bias. However, residual bias cannot be fully eliminated. Second, the size of the sample utilized in this study might impede the ability to generalize the results to a broader context. The small number of MRI (−) PCa (+) cases (*n* = 10) limits the statistical power of our NPV calculations, including the 100% NPV for PV > 55.25 mL, PSAD < 0.100 ng/mL^2^, and PSAD^adj^ < 7.24 ng/mL. These thresholds should be interpreted cautiously until validated in larger multicenter cohorts. Third, our study lacks of a follow‐up protocol for patients with negative biopsies, especially those with high‐risk predictors. Such a protocol could have helped determine PCa detection rates through repeat biopsies.

## 6. Conclusions

The incidence of csPCa in Chinese men with negative MRI is 8%. The NPV of MRI for csPCa is 92%. We posit that higher prostate volume, lower PSAD, or lower PSAD^adj^ thresholds can be used with negative MRIs in Chinese men to maintain appropriate NPV. Of note, negative MRI patients with prostate volume > 55.25 mL, PSAD < 0.100 ng/mL^2^, or PSAD^adj^ < 7.24 ng/mL can potentially avoid biopsies, given their 100% NPV. Yet, validation in other Chinese centers and prospective follow‐ups are required to confirm the safety and efficacy of these thresholds.

## Ethics Statement

The studies involving humans were reviewed and approved by the Ethical Committees of Beijing Tsinghua Changgung Hospital (Approval No. 2024‐012‐01). The studies were conducted in accordance with the local legislation and institutional requirements. Due to the retrospective nature of the study, the Ethical Committees of Beijing Tsinghua Changgung Hospital waived the need to obtain informed consent.

## Disclosure

All authors reviewed and approved the manuscript.

## Conflicts of Interest

The authors declare no conflicts of interest.

## Author Contributions

Fangming Wang, Yan Zhang, and Jianxing Li designed the study. Fangming Wang, Meng Fu, Haifeng Song, and Jianxing Li performed the prostate biopsy. Fangming Wang, Yan Zhang, Meng Fu, Yuzhe Tang, Haifeng Song, Gang Zhang, and Boxing Su analyzed the data. Fangming Wang and Yan Zhang wrote the manuscript. Fangming Wang, Yan Zhang, and Jianxing Li revised the manuscript.

Fangming Wang and Yan Zhang have contributed equally to this work.

## Funding

This work was supported by grants from Beijing Tsinghua Changgung Hospital Fund (12025C01015) awarded to Fangming Wang.

## Data Availability

The data that support the findings of this study are available on request from the corresponding authors. The data are not publicly available due to privacy or ethical restrictions.
